# Low rate of asymptomatic carriage and salivary immunoglobulin A response to Group A Streptococci in the healthy adult population in Finland

**DOI:** 10.1007/s00430-022-00750-9

**Published:** 2022-09-02

**Authors:** Emilia Lönnqvist, Kirsi Gröndahl-Yli-Hannuksela, Vuokko Loimaranta, Jaana Vuopio

**Affiliations:** 1 Institute of Biomedicine, University of Turku, MedisiinaD, Kiinamyllynkatu 10, 20520 Turku, Finland; 2 Institute of Dentistry, University of Turku, Turku, Finland; 3 Clinical Microbiology Laboratory, Turku University Hospital, Turku, Finland

**Keywords:** Streptococcus pyogenes, Saliva, Secretory IgA, Carrier state

## Abstract

*Streptococcus pyogenes,* also called group A streptococcus (GAS), is a human pathogen causing a wide range of infections ranging from mild tonsillitis to severe, life threatening conditions such as bacteraemia, necrotizing fasciitis, and streptococcal toxic shock syndrome. GAS may also colonise the oropharynx without causing any signs of disease which is known as asymptomatic carriage. This study aims to investigate IgA responses against GAS and oral streptococci from saliva samples collected from healthy Finnish adults. In addition, asymptomatic throat GAS carriage was studied. The study participants consisted of healthy adult volunteers who provided one saliva sample, a throat swab, and a background questionnaire. Total salivary IgA, and GAS specific IgA were analysed from the saliva samples using enzyme-linked immunosorbent assays (ELISA) and the results were compared to oral streptococci specific IgA levels. Asymptomatic GAS throat carriers were identified by bacterial culture, and the isolates were *emm* typed. Samples from a total of 182 individuals were analysed. The median salivary IgA concentration was 62.9 µg/ml (range 17.3–649.9 µg/ml), and median GAS and oral streptococcal specific IgA concentrations 2.7 and 3.3 arbitrary units (AU, range 1.4–7.4 AU and 1.6–12.0 AU), respectively. Three individuals with asymptomatic GAS throat carriage were identified.

## Introduction

*Streptococcus pyogenes*, also called group A streptococcus (GAS), is a gram-positive β-haemolytic bacterium that colonises epithelial and mucosal tissues of the human throat. The most common presentation of GAS infection is pharyngitis, although the spectrum of GAS disease is wide and includes also severe infections such as bacteraemia, necrotizing fasciitis, and streptococcal toxic shock syndrome [1]. GAS spreads through droplets or by contact, and therefore the oropharynx is the main point of entry into the human body. It has been estimated that globally, GAS cause over 600 million pharyngitis cases annually [2]. GAS can also colonise the oropharynx without causing any signs of disease; this is known as asymptomatic carriage. The GAS carriage rate among children can be over 10% [3], whereas in adults the reported carriage rate is much lower [4]. However, studies on asymptomatic throat carriage of GAS in adults are scarce.

Saliva is a major component in the oral cavity and plays an important role in oral homeostasis and the defence against pathogens. Saliva composition varies individually and is greatly affected by the salivary flow rate i.e. the volume of secreted saliva over time [5]. The main antibody class in saliva is secretory immunoglobulin A (sIgA), which is produced in plasma cells in the salivary glands [6]. sIgA is part of the first line defence against pathogens encountered in the oropharynx. sIgA prevents the pathogen from adhering to host tissues, and binds the bacteria to mucins, which increases bacterial clearance through saliva excretion [6, 7].

Despite of the several decades of investigations on pathogenesis of GAS and the fact that GAS produces a vast number of virulence factors, there is no clear understanding of the correlate of protection against GAS [8]. This is one of the reasons hindering the vaccine development against GAS. In addition, the role of asymptomatic carriage or asymptomatic recurrent infections on the immune responses against GAS is not well understood [9, 10]. Despite the importance of sIgA in the microbial control of the oral cavity, the GAS specific immune response in saliva has, to our knowledge, not been extensively studied in the healthy population or in individuals with GAS throat carriage or GAS infections. Previous studies have mainly focused on salivary antibody responses against oral streptococci or after oral immune-stimulants treatment [11–16]. Very recently, a human challenge model of GAS pharyngitis investigated the immunological responses in saliva samples, however, antibodies were not measured [17].

The aims of this study were to investigate the total and GAS specific salivary IgA concentrations in healthy Finnish adults. In addition, we aimed to determine the prevalence of asymptomatic GAS throat carriage in the study population.

## Materials and methods

### Ethical considerations

The study was performed according to the Declaration of Helsinki. Participation in the study was voluntary and participants gave their informed written consent. All data and samples collected during the study were handled pseudonymised and confidentially. This study has been approved by the Ethics Committee, Hospital District of Southwest Finland (80/1801/2019 §323).

### Study population

Healthy volunteers were recruited among students and personnel at the University of Turku, Finland, from November 2019 to March 2020. Volunteer recruitment to the study was cancelled prematurely due to the COVID-19 pandemic. The study participants were asked to provide a saliva sample and a throat swab. In addition, subjects answered a questionnaire, in order to collect information about age, sex, and possible diagnosed GAS pharyngitis during the past 6 months. The inclusion criteria were: age ≥ 18 years and no signs of GAS pharyngitis i.e. sore throat and fever. Exclusion criteria were: any self-reported signs of pharyngitis i.e. sore throat and fever, no current or previous (last 4 weeks) use of antibiotics, or diagnosed IgA deficiency.

### Sample collection and processing

The participants were asked not to eat, drink, or use dental hygiene products at least one hour before sampling. Throat samples were collected from the tonsils with standard technique using sample collection cotton swabs. Samples were cultured immediately on agar plates (TSA with 5% sheep blood, BD, USA) and incubated 16–20 h at + 35 °C with 5% CO_2_. β-haemolytic, catalase negative isolates were analysed with the Streptex agglutination test (Remel Europe Ltd, Kent, UK) to identify Lancefield group A streptococci and stored at − 80 °C. Isolates confirmed as GAS were *emm* typed according to the CDC standardised protocol (https://www.cdc.gov/streplab/groupa-strep/emm-typing-protocol.html).

Saliva samples were collected by chewing a synthetic swab for 1 min (Salivette, Sarstedt, Germany). To separate the saliva from the swab, the collection tubes with the swab were centrifuged (1000 × g, 2 min) and the saliva was transferred to new, sterile tubes and stored at − 80 °C until further analysis. Saliva collection and separation method used yields mainly to serous fraction of the saliva. The volume of collected saliva was recorded.

### Determination of IgA in saliva

To determine the concentration of IgA in the saliva samples, we set up an enzyme-linked immunosorbent assay (ELISA) adapted from Sonesson et al. [18]. Shortly, 100 µl of coating antibodies (5 µg/ml goat anti-human IgA (Merck, I0884) in 0.05 M carbonate bicarbonate buffer (Merck C3041, pH 9.5 at 25 °C) were added to the wells of a microtiter plate (Nunc-Immuno Maxisorp, ThermoFisher) and incubated overnight at room temperature. Plates were washed 3 times with PBS + 0.05% Tween20 (PBST) and blocked with PBST + 1% bovine serum albumin (BSA) 100 µl/well for 1 h at + 37 °C. To generate a standard curve, a serial dilution of IgA from human serum (Sigma, I4036) in PBST was used (1, 0.5, 0.25, 0.125, 0.0625, 0.03125 and 0.0156 µg/ml). Saliva samples were centrifuged (16,000 × *g,* 5 min) to remove precipitates formed after freezing the samples, and diluted 1:500, 1:1000, 1:3000, and 1:5000 in PBST. After the blocking step, the plate was washed as described previously and 100 µl of each standard and sample was added in duplicate and incubated for 1 h at + 37 °C. The plate was washed as previously, 100 µl of the conjugated detection antibody (goat anti-Human IgA − Alkaline Phosphatase (Merck, A9669), diluted 1:30 000 in PBST) was added and incubated for 1 h at + 37 °C. Plates were washed as previously and 200 µl of phosphatase substrate (pNPP, Merck S0942, diluted to 1 mg/ml in diethanolamine-MgCl_2_ buffer, Reagena Finland) was added and incubated for 30 min at + 37 °C. The reaction was stopped by adding 50 µl 3 M NaOH to each well and the optical density was measured at 405 nm (OD_405_). The IgA concentration in the saliva samples was extrapolated from the generated standard curve. Saliva from 10 healthy volunteers were pooled and used for validation. The intra- and inter assay coefficient of variation were 2.8% and 12.5%, respectively.

### Determination of GAS and oral streptococcal specific sIgA

#### Bacterial cultures and preparation of whole cell lysates

We developed an ELISA based method to determine the concentration of GAS specific IgA in saliva. Whole bacterial cell lysates were used as coating antigen. We prepared a pooled lysate of three *S. pyogenes* strains: MGAS5005 (*emm*1, ATCC strain BAA-947), MGAS6180 (*emm*28, ATCC strain BAA-1064), and MGAS27520 (*emm*89, a Finnish clinical isolate kindly acquired from the Finnish Institute for Health and Welfare) [19]. These strains represent the most common *emm* types in bacteraemic GAS infections in Finland at current [20, 21]. As a control, another pooled lysate was prepared from three common oral streptococcus species: *S. mutans* (NCTC 10,449), *S. salivarius* (ATCC 25,975), and *S. sanguinis* (NCTC 10,904) [22].

All bacterial strains were cultured in Todd Hewitt broth supplemented with 0.2% yeast extract (Todd Hewitt yeast, THY) overnight at + 37 °C with 5% CO_2_. Cultures were centrifuged (2800 × *g*, 15 min) and the bacterial pellet was washed twice with PBS. The cells were diluted to a final concentration equivalent of OD_600_ 0.5 and pooled. The lysates were heat inactivated at + 85 °C for 45 min and stored at − 20 °C.

#### ELISA protocol for GAS and oral streptococcal IgA

An ELISA protocol for detection of sIgA specific for GAS and oral streptococci was modified from the protocol for IgA described above. In brief, coating was done by adding either GAS or oral streptococci whole cell bacterial lysate (see above) to microtiter plate wells and incubate overnight at + 4 °C. Wells were washed and blocked as described above. Saliva samples were diluted 1:10 in PBST. 100 µl of the samples were added in duplicate and incubated for 2 h at + 37 °C. After washing 3 times, 100 µl of conjugated detection antibody (anti-Human IgA − Alkaline Phosphatase, 1:10,000) was added and incubated for 1 h at + 37 °C. Plates were washed and 200 µl of phosphatase substrate was added and incubated for 30 min at + 37 °C. After stopping the reaction (3 M NaOH) the absorbance was read (405 nm). The specific IgA response was calculated in arbitrary units (AU) by dividing the value of OD_405_ of the individual sample with OD_405_ of a blank (PBST) sample [18]. One pooled saliva sample was used as control for assay validation, (mean OD_405_ for GAS = 0.425 and for oral streptococci = 0.883). The intra and inter assay coefficient of variation were 2.8% and 13.9% for GAS specific IgA, respectively and 3.7% and 11.4% for oral streptococcal IgA, respectively.

#### Statistical analysis

The IgA concentrations between different groups were compared by Wilcoxon/Kruskal–Wallis Tests. P values < 0.05 were considered statistically significant. Spearman’s rank correlation coefficient was used to evaluate the specific relationship between GAS and oral streptococcal IgA and Pearson correlation to evaluate the relationship between saliva volume and saliva IgA concentration. Statistical analyses were performed using JMP Pro 14 (SAS Institute Inc., USA). Figures were prepared using GraphPad Prism 8 (GraphPad Software, USA). Coefficient of variation was used for inter-assay and intra-assay validation.

## Results

### Sample collection

A total of 197 individuals were recruited and provided both a throat swab and a saliva sample. After excluding 15 subjects who did not meet the inclusion criteria (subjects reported current throat pain or fever), 182 subjects were included. Answers to the questionnaire were missing from one individual. The age range of the participants was 19 to 55 years (n: 181, median 23 years). 98 (53.8%, 98/181) were women (range 19–55 years, median 23 years) and 83 (45.6%, 83/181) were men (range 20–44 years, median 23 years). Two individuals reported a previous GAS pharyngitis diagnosis within the past 6 months.

The median volume of saliva collected was 1.1 ml (range 0.42–2.99 ml). All saliva samples were visually examined for blood contamination and none were observed.

### Asymptomatic GAS carriage

Three individuals (1.6%, 3/182) were identified as asymptomatic throat GAS carriers. Two of the carriers were women and one man. *Emm*-types of the GAS isolates identified were *emm*89.0, *emm*28.0, and *emm*164.6.

### Total salivary IgA

**Fig. 1:**
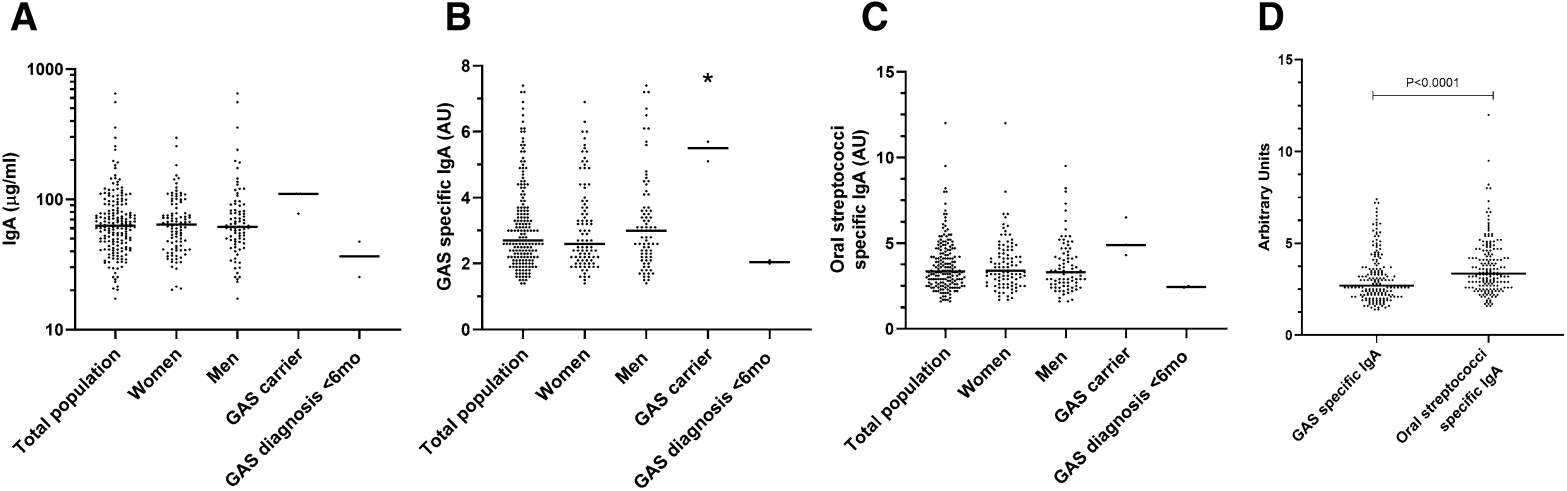
Salivary IgA concentrations in the study population (*n* = 182) **A** IgA concentrations (µg/ml) in saliva. **B** Salivary GAS specific IgA in arbitrary units (AU). **C** Salivary oral streptococci specific IgA in arbitrary units. **D** Salivary GAS specific IgA and oral streptococci specific IgA expressed in AU. Each data point represents the IgA concentration of one study subject. The horizontal line indicates the value of the median concentration of the population. *There was a statistically significant difference in GAS specific IgA between asymptomatic GAS carriers and non-carriers (*P* = 0.0131, not shown) and between GAS and oral streptococci specific AU levels (*p* < 0.0001). GAS: Group A *Streptococcus*, OD: optical density, oral strep: oral streptococci (*S. salivarius*, *S. mutans*, and *S. sanguinis*)

The median salivary IgA concentration in the study population was 62.9 µg/ml, and there was a huge variation between individuals (Table 1, Fig. 1A). The total salivary IgA concentration did not differ significantly between genders, asymptomatic GAS-carriers and non-carriers or those with a GAS diagnosis within the past 6 months (Fig. 1A). IgA concentrations and the saliva volume were inversely correlated (*r* = − 0.26/*r*^2^ = 0.06).

**Table 1:** Median salivary IgA concentrations in the study population (*n* = 182)

	*n*	IgA (µg/ml) (range)	GAS specific IgA (AU) (range)	Oral strep. specific IgA (AU) (range)
Total population ^a^	182	62.9 (17.3–649.9)	2.7 (1.4–7.4)	3.3 (1.6–12.0)
Female	98	64.1 (20.3–297.2)	2.6 (1.4–6.9)	3.4 (1.7–12.0)
Male	83	61.5 (17.3–649.9)	3.0 (1.4–7.4)	3.3 (1.5–9.5)
GAS carrier	3	110.4 (77.5–110.4)	5.5^b^ (5.1–5.7)	4.9 (4.3–6.5)
Non-GAS carrier	179	62.7 (17.3–649.9)	2.7^b^ (1.4–7.4)	3.3 (1.6–12.0)
Pharyngitis within 6 months	2	36.4 (25.3–47.4)	2.1 (2.0–2.1)	2.5 (2.4–2.5)
No pharyngitis within 6 months	179	62.9 (17.3–649.9)	2.7 (1.4–7.4)	3.4 (1.6–12.0)

^a^ One individual did not return the study questionnaire. ^b^ There was a significant difference in GAS specific IgA concentration between asymptomatic GAS carriers and non-carriers (*P* = 0.0131)

### Streptococci specific IgA

The salivary IgA concentrations against GAS and oral streptococci were investigated and expressed as arbitrary units (AU). The median GAS specific IgA concentration was 2.7 AU (Table 1, Fig. 1 B). The 95th percentile was reached at 6.1 AU, and nine individuals had a GAS specific IgA level above this value. The median volume of collected saliva from these subjects was 0.98 ml (range 0.42–1.6 ml). The asymptomatic GAS carriers had significantly higher GAS specific IgA concentrations in their saliva compared to the non-carriers (*P* = 0.01, Fig. 1 B). All GAS carriers had GAS IgA values below the 95th percentile. GAS specific IgA levels for the GAS carriers were 5.7 AU (*emm*28.0 carrier), 5.5 AU (*emm*89.0 carrier), and 5.1 AU (*emm*164.6 carrier). There was no statistical difference in GAS specific IgA concentrations in relation to gender or previous GAS diagnosis.

The median oral streptococcal specific IgA concentration was 3.3 AU (Table 1, Fig. 1C). The 95th percentile was at 6.5 AU. The concentration of oral streptococci specific IgA was significantly higher than the GAS specific (*P* < 0.0001, Fig. 1D). There was no statistical difference in the oral streptococci specific IgA concentrations between genders, previous GAS diagnosis, nor between GAS carriers and non-carriers.

Six out of nine individuals with GAS IgA AU higher than the 95th percentile also possessed oral streptococcal specific IgA levels above the 95th percentile. The median concentration of total salivary IgA in these six subjects was 176 µg/ml.

**Fig. 2:**
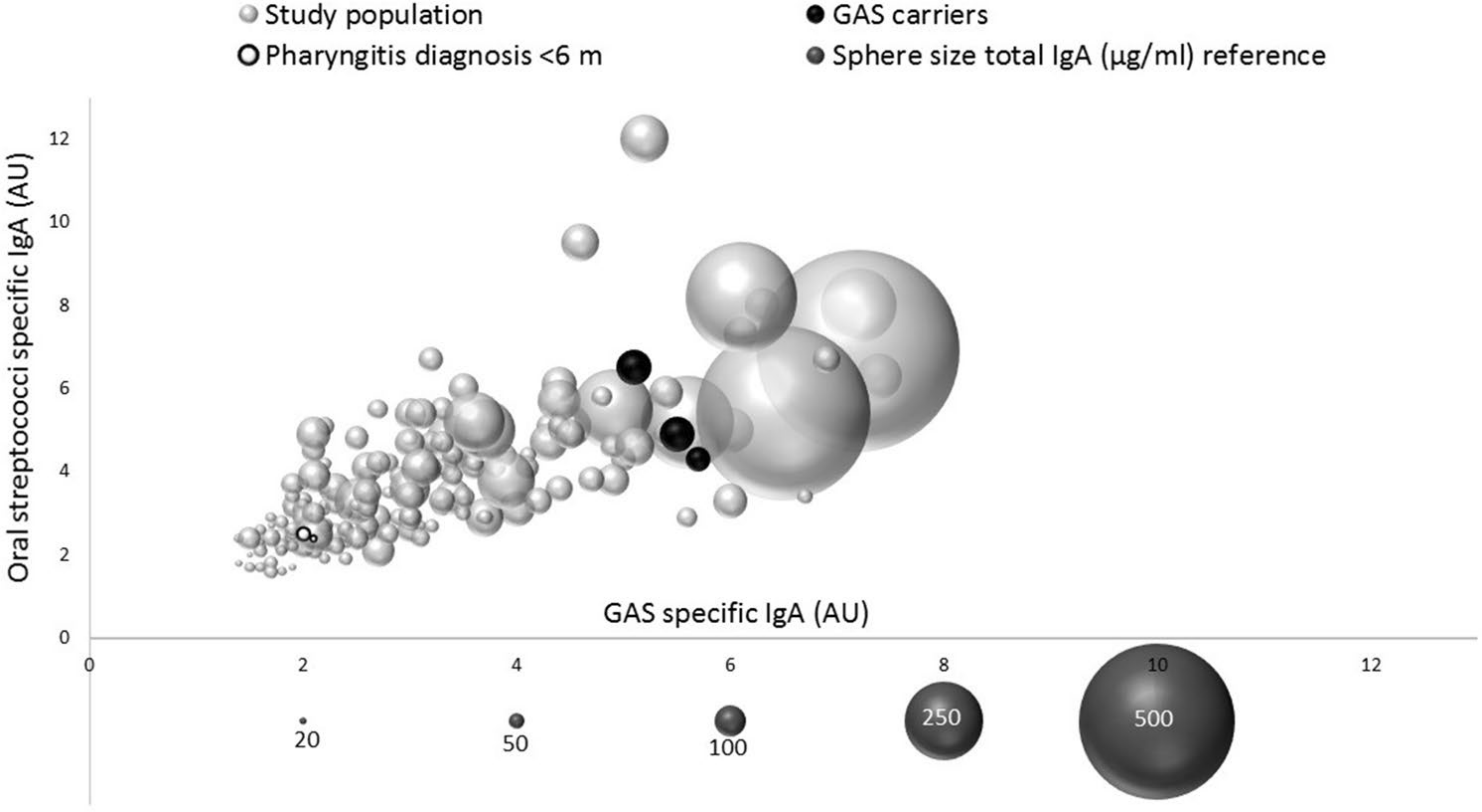
The relationship between GAS (x-axis) and oral streptococci (y-axis) specific salivary IgA in arbitrary units (AU) and total salivary IgA concentration (diameter of the sphere). Each sphere represents one individual (*n* = 182). The three individuals identified as asymptomatic GAS carriers, are indicated in black colour. Individuals with positive GAS diagnosis within the last 6 months are indicated with white sphere with black outline (*n* = 2), all other study subjects in light grey (*n* = 177). Lower axis of the Figure: dark grey spheres serve as a size reference for the total salivary IgA concentrations (µg/ml)

The concentrations between GAS specific IgA and oral streptococci specific IgA showed a positive correlation (*r* = 0.719). In addition, we observed a moderate positive correlation between total salivary and GAS specific IgA levels (*r* = 0.680) and between total salivary and oral streptococci specific IgA (*r* = 0.675) (Fig. 2). The two individuals with previous GAS infection diagnosis expressed low levels of all measured IgA (total, oral streptococci and GAS specific IgA).

## Discussion

In this study, we investigated the salivary concentrations of total and GAS specific IgA in 182 healthy adult volunteers in Southwest Finland. In addition, we screened the study participants for asymptomatic GAS throat carriage to identify possible differences in salivary IgA concentrations between carriers and non-carriers. To the best of our knowledge, the significance of GAS specific IgA in the saliva of healthy adults has not previously been studied in a population of this size.

Detection of anti-streptolysin O and anti-DNase B antibodies from sera is used in clinical laboratories for diagnostics of post-streptococcal diseases such as rheumatic heart disease [23]. However, the immune responses have been found to vary greatly between asymptomatic and symptomatic GAS pharyngeal carriage [9]. The salivary immune responses to GAS, in both infected and in healthy adults, have been studied less extensively. Anti-GAS sIgA levels in human saliva have been found to increase after treatment with oral immune-stimulants containing bacterial components [11, 12].

None of the nine individuals who had GAS specific IgA levels higher than 95th percentile was an asymptomatic GAS carrier. The saliva volume from these subjects was similar with the median of the whole cohort. This indicates that the high anti-GAS IgA observed may not be explained by saliva secretion level. We used a pooled lysate of three different *emm* types as the coating antigen, however, we do not possess any more detailed information about each individual’s previous history of GAS disease.

Interestingly, we found that the two individuals with recent GAS pharyngitis (within 6 months), produced a low amount of all measured IgA (total, GAS and oral streptococcal specific). This discrepancy could possibly be explained by previous antibiotic treatment. It has been shown that children with recurrent tonsillitis have lower levels of saliva IgA, and continuous antibiotic treatment may cause decreased IgA levels [24–26]. However, we do not possess any information about these individuals’ medical history other than the information provided in the questionnaire. Two individuals produced relatively high total salivary IgA concentrations (> 500 µg/ml). Salivary IgA concentrations show large individual variation and are dependent, for example, on salivary flow rate [25]. In this study, saliva was collected by chewing a swab for 1 min, and no information of the flow rate is available. The highest IgA concentrations were, however, measured from samples with lowest saliva recovery indicating reduced salivary secretion in these individuals.

The observed prevalence of asymptomatic GAS throat carriage (1.6%) rate was similar with previous studies from Finland [27] and in OECD countries [4]. We identified a difference in GAS specific IgA levels between asymptomatic carriers and non-carriers. However, there were only three GAS carriers observed which is not sufficient for making any valid conclusions. This is still an interesting finding and further studies on this are encouraged. It would be of interest to compare the GAS specific IgA saliva concentration from GAS tonsillitis patient samples with asymptomatic carriers.

We acknowledge the fact that there are several limitations to our study. For detection of specific streptococcal IgA in saliva, we used a pooled cell lysate of three different GAS *emm* types to compensate for variations between isolates. The *emm* types were chosen based on the most prevalent *emm* types among invasive GAS infections in Finland. However, the *emm* type distribution might be different among asymptomatic throat carriers, which could potentially lead to decreased detection of GAS specific IgA from other *emm* types not included in our coating lysate. Antibodies against different streptococcal species show cross reactivity [28] so for comparison, we used a pooled lysate of three oral streptococcal species to determine the specificity of the assay. A positive correlation was observed between the pooled lysates which may indicate some cross reactivity between the IgA specific for GAS and oral streptococci. The usage of monomeric IgA as a standard might affect the overall IgA measurements as well. For practical reasons our selection of oral streptococcal species was limited, but strains were selected to represent three main group of oral streptococci, Mutans-, Mitis-, and Salivarius-groups. Due to the huge diversity of oral streptococci it should be noted that these strains do not represent the complete antigenic variability of the oral streptococcal flora, which should be addressed in future studies. The medical history of the study participants in regards to any potential history of GAS disease, and their general oral health was not available. However, this would be important to take into consideration during future studies, to account for the potential effect oral and dental infections may have on the general IgA levels in the saliva.

To conclude, we have investigated total, GAS and oral streptococcal specific IgA levels in saliva in healthy Finnish adults. We identified three asymptomatic GAS throat carriers and observed large individual variations in GAS specific IgA levels, as well as sIgA concentration. Interestingly we found that asymptomatic GAS carriers have higher levels of GAS specific IgA, while those with recently diagnosed GAS tonsillitis have low levels of both sIgA and GAS specific IgA. Further studies are needed to enlighten the relationship between asymptomatic GAS carriage and the salivary immune response.

## Data Availability

The datasets generated during and/or analysed during the current study are available from the corresponding author on reasonable request.
